# The emotion system promotes diversity and evolvability

**DOI:** 10.1098/rspb.2014.1096

**Published:** 2014-09-22

**Authors:** Jarl Giske, Sigrunn Eliassen, Øyvind Fiksen, Per J. Jakobsen, Dag L. Aksnes, Marc Mangel, Christian Jørgensen

**Affiliations:** 1 Department of Biology, University of Bergen, PO Box 7803, 5020 Bergen, Norway; 2 Hjort Centre for Marine Ecosystem Dynamics, Bergen, Norway; 3 Center for Stock Assessment Research and Department of Applied Mathematics and Statistics, University of California, 1156 High St., Santa Cruz, CA 95064, USA; 4 Uni Computing, Uni Research, Thormøhlensgate 55, 5008 Bergen, Norway

**Keywords:** emotion system, trait architecture, diversity, convergent evolution, evolutionary innovation, evolvability

## Abstract

Studies on the relationship between the optimal phenotype and its environment have had limited focus on genotype-to-phenotype pathways and their evolutionary consequences. Here, we study how multi-layered trait architecture and its associated constraints prescribe diversity. Using an idealized model of the emotion system in fish, we find that trait architecture yields genetic and phenotypic diversity even in absence of frequency-dependent selection or environmental variation. That is, for a given environment, phenotype frequency distributions are predictable while gene pools are not. The conservation of phenotypic traits among these genetically different populations is due to the multi-layered trait architecture, in which one adaptation at a higher architectural level can be achieved by several different adaptations at a lower level. Our results emphasize the role of convergent evolution and the organismal level of selection. While trait architecture makes individuals more constrained than what has been assumed in optimization theory, the resulting populations are genetically more diverse and adaptable. The emotion system in animals may thus have evolved by natural selection because it simultaneously enhances three important functions, the behavioural robustness of individuals, the evolvability of gene pools and the rate of evolutionary innovation at several architectural levels.

## 1. Introduction

In evolutionary ecology, there has been a focus on finding the optimal phenotype for a given environment, with Optimal Foraging Theory [1,2], Life History Theory [3,4], Game Theory [5,6] and State-Dependent Behavioural and Life History Theory [7,8] as the major methodologies. However, with few exceptions (e.g. [8,9]), these generally do not consider the fitness of sub-optimal phenotypes, the impacts of adaptive phenotypes on the genome and gene pool, or the constraints evolutionary pathways make on the adaptive solution [10].

There are at least two evolutionary obstacles to arriving at the optimal phenotype: the evolution of the gene pool and the formation of the phenotype from the genotype. First, the fitness landscape may not have a single peak that is easily accessible from all starting points. A rugged or holey landscape with many solutions creates a path-dependence [9,11]—a historical contingency—in the process of adaptation. In addition, if frequency-dependence is important in selection, then the fitness landscape changes according to the present state of the gene pool, such that the location and surroundings of peaks and paths between them may be constantly changing. These factors suggest that the process of adaptation, even in the same physical environment, may end up at different peaks in the fitness landscape. Second, according to the ‘phenotypic gambit’ [12], the assumption that natural selection leads to the optimal phenotype further assumes a direct correspondence between an unconstrained genotype and the behaviour or trait that is directly relevant for fitness [13]. Optimization approaches thus usually overlook all the mechanisms between the strength of selection and the behaviour or other phenotypic trait assumed to be optimal, for example genetic coding, sensing, cognition and decision-making [14–16].

While behavioural ecologists have used the phenotypic gambit as an argument for what to expect at the phenotypic level [12,17], one can also ask about the consequences for the gene pool of adding mechanistic layers between the genotype and the phenotype [18–20] and the consequence in terms of predictability and diversity for these intermediary layers themselves to be under selection [21–23]. We investigate this question in an idealized model of the emotion system [24] that links genes to phenotype and study evolutionary adaptation in environments that include possibilities for age-, state-, density- and frequency-dependent adaptation and behaviour. This allows us to simultaneously investigate whether trait architecture or environmental factors alter the tendency of gene pools to arrive at the same peaks in the fitness landscape, the resulting phenotypic diversity in the adapted populations and the characteristics of gene pools of adapted populations with trait architecture.

We find that a dominant trait architecture in higher animals, the emotion system, is itself sufficient for generating diversity, and that neither environmental variation nor complexity will prevent the population from arriving at predictable phenotypes. We also show that the predictability decreases as one moves from life-history traits to gene pools. Our results indicate that trait architecture gives individuals the flexibility to respond appropriately to new situations in the short term (a lifetime), but also that architecture has long-term evolutionary consequences as it leads to diverse gene pools with high adaptability to a wide range of new challenges.

## 2. Material and methods

The emotion system is central in converting sensory information via decision-making into behaviour, at least in vertebrates [25–28]. It is a system in a behavioural sense, but it is not a neurobiological unit. While the Euler–Lotka equation [29] and later the method of dynamic optimization [8] have been proposed as models for the ‘common currency’-mechanism for evolutionary adaptations by natural selection, the emotion system acts as a proximate common currency mechanism: it is evolutionarily adapted to integrate and evaluate information of widely different usage for the organism.

**Figure 1.**
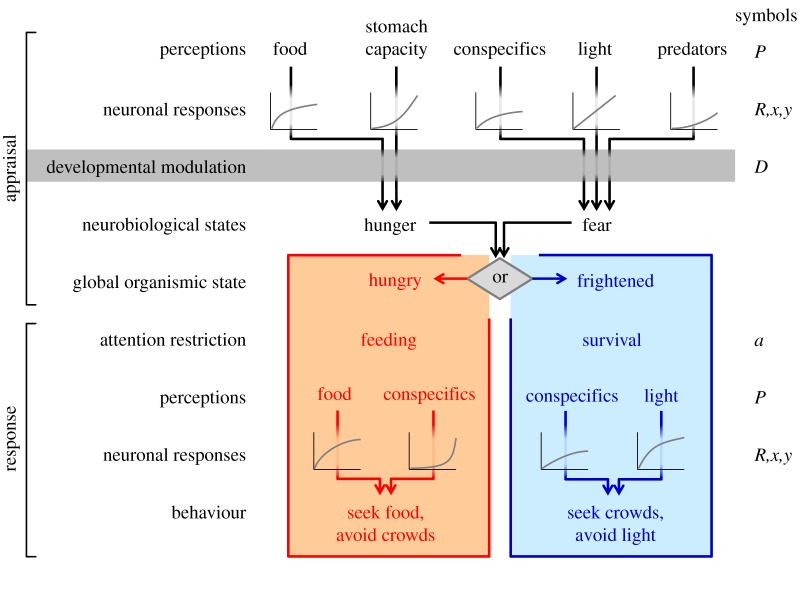
How emotions translate perception stimuli into behavioural responses in the model of Giske *et al*. [24] and in this paper. Each type of emotional stimuli contributes to an emotional appraisal through neuronal response, developmental modulation and competition among hunger and fear. The strength of each neuronal response *R* is individual and depends on the strength of the perception *P* and two genes specific to each neuronal response (*x* and *y*): *R* = (*P*/*y)^x^*/⌊1 + (*P*/*y*)*^x^*⌋. This equation gives a curve which, depending on the alleles of *x* and *y* in this individual, can be concave, sigmoidal, nearly linear or convex, as illustrated in the neuronal response cartoons in this figure and shown in figure 5. Internal signals related to development *D* are also individual and may amplify the strength of inputs to hunger (*D*) or fear (1 – *D*) over the other. The strengths of the competing neurobiological states in the hunger and fear survival circuits are then 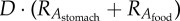 and 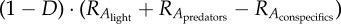, respectively. (The subscript *A* indicates that these are neuronal responses involved in the emotional appraisal.) The emotional appraisal ends with the stronger of these determining the global organismic state of the organism [28]. The emotional response is specific to the global organismic state and includes physiology and behaviour. The physiological response to this emotional appraisal is an attention restriction of the organism. In the processing of its behaviours, it thus re-evaluates a subset of its sensory information in its current depth *z* and the immediate depths above and below. Hungry individuals (neuronal response subscript *H*) will value food as positive and competitors as negative and ignore other information: 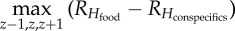. Frightened individuals (subscript *F*) will regard light as negative and conspecifics as positive: 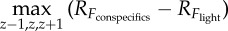. When the relevant behaviour is executed, the animal starts over on top with new emotional stimuli. Adapted from [24].

We use a model of a fish population where the trait architecture includes genetics, physiology, emotion, behaviour and reproduction with inheritance [24]. In this model, perception, neuronal responses and developmental modulation are influenced by the genome and determine the individual's ‘global organismic state’ [28] which restricts its attention [30,31] and constrains its behavioural choices [28] (figure 1).

Our model describes fish in a pelagic environment, so that the behavioural alternatives are to move one level up or down or to remain at the same depth. Generations are non-overlapping and last for seven diel cycles of continuous surface light change. (Hence, we have condensed a year to 7 days.) There are 200 time steps per day where the fish determines its depth according to the processes illustrated in figure 1. Light decays with depth, impacting both predation risk and feeding opportunity, and thus creates a temporally and spatially variable environment [32,33]. Crowding reduces individual mortality risk but also generally increases competition for food [34–36]. See detailed descriptions in the electronic supplementary material of [24]. (Although unseen by most people, mesopelagic fishes are the most abundant fishes in the ocean [37].) The emotion model considers two attention-competing survival circuits [28]. The dominating circuit sets the organism in a global organismic state, which in our case is fear or hunger. Individuals whose emotion system (figure 1) has made them afraid, regard light as a risk enhancer and conspecifics as risk dilutors, while hungry fish are attracted to food and regard conspecifics as competitors [24].

The form in which genes impact the emotion system is central to the model. Thus, while all hungry individuals regard conspecifics as negative in the evaluation of the quality of a potential resource (figure 1), some may pay little attention to conspecifics while others may strongly avoid them [24]. We assume that there are two genes in each of the nine neuronal response functions that link a sensory perception of the environment or of the state of the organism to emotions and behaviours in the model (figure 1). Further, the organism has one sex-determination gene and four genes that modulate the impact of development (through body mass) on fear and hunger. Hence, each haploid fish has a unique set of alleles of 23 genes inherited from its parents. The two genes in a neuronal response together form a chromosome, which is subject to mutation but not recombination. The sex-determination gene and the four developmental genes are located on one chromosome. As a consequence, developmental modulation may become sex-differentiated, while a neuronal response chromosome will alter between being in female or male individuals. Selection differs among the sexes as a female who survives until the last time step of a generation produces eggs in proportion to her body mass and then chooses the largest of three randomly encountered surviving males as father for her eggs. Hence, there is larger variation in parenthood in males, driving selection for larger body mass via the genes for developmental modulation, thus less fear and higher mortality. Examples are the spikes in mortality in males, explained in fig. 4 in [24]. The model is explained in detail according to the ODD (overview, design concepts and details) protocol [38,39] in [24].

We studied the effects of environmental and organismal complexity in four scenarios (table 1), ranging from no environmental variation in time and space (‘game only’) and no density- or frequency-dependent processes (‘no game’), via an environment that is exactly the same in each generation (‘repetitive environment’), to rich environmental variation and density-dependency (‘full’ scenario). For each scenario, we ran 30 simulations over 20 000 generations, initiated with 10 000 individuals with random alleles. The mutation risk of one gene in one individual is 0.001. Ninety per cent of mutations are to one of the nearest alleles, and 10% are to a random allele. This sums to 200 000 mutations per gene over a simulation. Behavioural and life-history phenotypes did not converge in 13 of the 30 simulations in the ‘game only’ scenario, even after 20 000 generations. We therefore also ran 10 simulations for more than 100 000 generations in the most extreme scenarios (‘game only’ and ‘full’).

**Table 1.** The four experimental scenarios.

scenario	features
full	food competition and predation risk dilution are density-dependent. Risk is also body-size dependent while feeding may be constrained by stomach capacity. Full intergenerational environment variation as described in table 2
repetitive environment	all generations experience the same standard environment of table 2. The random medium-term environmental fluctuations in prey density, predation risk and light intensity described under fluctuating environments in table 2 are removed. Habitat profitability is density-dependent as in full scenario. Predator schools attack as in full scenario
game only	uniform distribution of prey and no light variation in time and space. Space variation is only caused by the location of the population. Time variation is only caused by predator schools (as in standard environments in table 2) and by body-size-dependent food demand and mortality risk (larger bodies more easily seen)
no game	full intergenerational environmental variability (table 2), but growth and survival are not impacted by presence of conspecifics. Random mating

In the ‘full’ scenario, we only used generations with ‘standard environment’ (table 2) to compare simulations. We collected the statistics of genotypes (details in the electronic supplementary material, figure S1) from all individuals in the 200 last ‘standard environment’ generations in each simulation. The ‘no game’ and ‘game only’ scenarios do not include this standard environment but have other environments that repeat each generation (table 1). For these scenarios, we used results from the 200 last generations. We measured non-genetic data at six fixed periods or points through life for each individual in the final generation, except for life-history data which were measured only at the end of the generation. Thus, for each scenario, our results are based on 150 000 observations of each sex-specific life-history parameter, 900 000 observations of other phenotypic parameters, 30 million observations of the sex-specific developmental genes and 60 million observations of all the other genetic parameters. We quantified within-population diversity for 88 individual variables for the 30 populations in each scenario by the average within-generation coefficient of variation (CV) over these 200 generations.

**Table 2.** Environmental variation within one generation in some of the scenarios.

environment	description
standard	diel light cycle which impacts detection of prey and vulnerability to predators. Vertical environment where light intensity fades off. A renewing prey population follows a fixed vertical migration pattern with ascent to surface in evening and descent in morning and with highest densities at the centre of distribution. A school of predatory fish attacks with same intensity four fixed times during life (see the electronic supplemented material, figures S2–S5)
fluctuating	frequent medium-term random fluctuations in prey density, mortality risk and light intensity, within ±20% of the standard value, each fluctuation lasting 20% of a generation
lower risk	mortality risk everywhere decreased by the same random factor throughout the generation, down to minimally 50% of standard
higher risk	mortality risk everywhere increased by the same random factor throughout the generation, up to maximally 150% of standard
deep food	no food in the five shallowest (out of 30) cells
shallow food	no food in the six deepest cells
less food	prey density everywhere decreased to 85% of standard
more food	prey density everywhere increased to 125% of standard
more food and higher risk	prey density and mortality risk everywhere increased to 125% of standard

We estimated evolutionary diversity among the populations within a scenario by interquartile range and total range of within-population averages for the same variables, after normalizing each population average against the average among populations. This standardized the variation relative to an average of 1. We excluded the 13 populations that did not arrive at the scenario-typical phenotypic solutions (i.e. the adaptive peak) in the ‘game only’ scenario from the analysis of variation within and among adapted populations.

## 3. Results

Phenotypic diversity evolved within and among all populations, and this diversity was lowest for life-history traits and increased as one moves via physiological and behavioural traits and global organismic state through to the genotype. In the following, we first describe the ‘full scenario’ as basis for comparison, and then relate it to the other scenarios to explain patterns of diversity in light of trait architecture.

### (a) Phenotypic convergence in scenarios with high fidelity to nature

**Figure 2.**
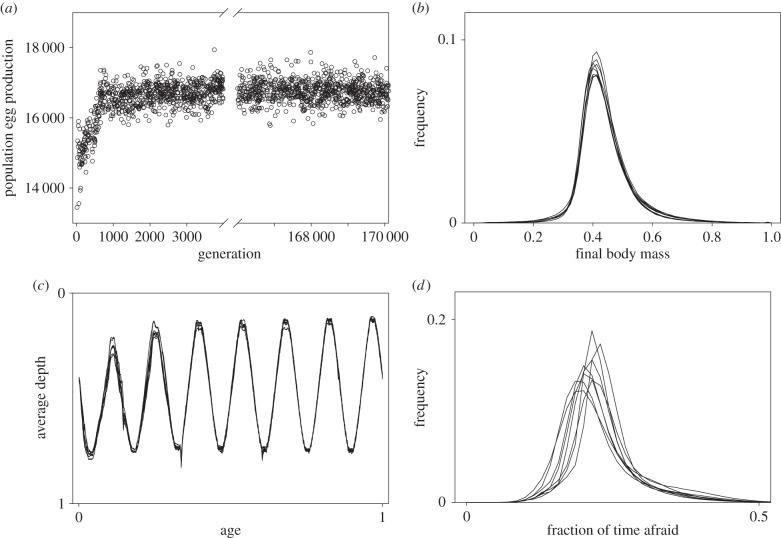
(*a*) Evolution of population egg production in one simulation in the ‘full scenario’. (*b*) Body mass at the end of a life cycle, (*c*) average depth through life and (*d*) population variation in tendency of being afraid in the same population, shown for females for every 20 000 generations, up to generation 160 000.

In the scenario with the highest fidelity to the situation of small planktivorous fish—the ‘full’ scenario with density-dependency, frequency-dependency and rich environmental variation—the evolving populations went through an initial phase of rapid adaptive changes, best seen as an increase in the egg production (figure 2*a*). This initial phase was usually shorter than 2000 generations but varied in length between simulations (electronic supplementary material, figure S2). After the initial phase, the allele frequencies still changed continuously, but this had little impact on total fecundity of the population or average phenotypic characters (figure 2). After this initial phase, all phenotypic characters (final body mass, mortality, crowding and average depth) converged across the simulations (figure 2 and the electronic supplementary material, figures S2–S5). Similar phenotypic convergence was seen in all simulations of all scenarios, except for the ‘game only’ scenario (electronic supplementary material, figures S2–S5).

Decreasing the environmental variation from ‘full’ to ‘repetitive’ (table 1) did not decrease variation within or between populations (electronic supplementary material, figures S2–S5). The ‘standard environment’ of the ‘repetitive’ scenario already contains diel and vertical environmental variation as well as density-dependent effects of the presence of conspecifics (table 2).

**Figure 3.**
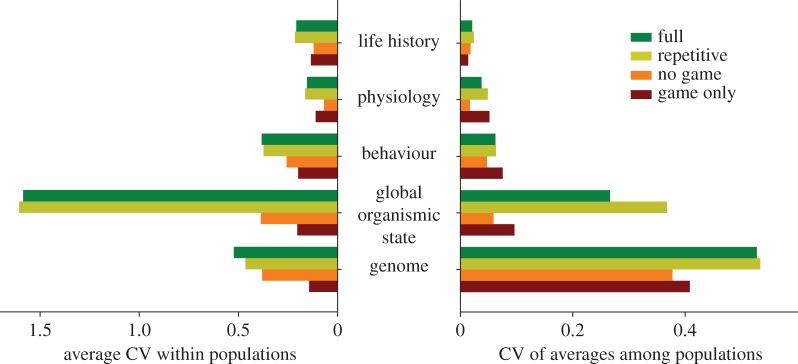
Genotypic and phenotypic diversity within and between scenarios. (*a*) Diversity within populations. Average within-population CV of all 88 parameters sorted into architectural level from genes to life history in each of the four scenarios. These parameters are detailed in the electronic supplementary material, figure S1, for all four scenarios. (*b*) Diversity between populations. CV among the 30 populations in each scenario of the within-population average of the same variables. (Online version in colour.)

The scenarios without density-dependence and frequency-dependence (‘no game’) and environmental variation (‘game only’, see below) had the lowest within-population variation in all parameters (figure 3). However, there was genetic, global organismic state and phenotypic variation both within and between all populations.

### (b) Slow or no convergence in the pure game scenario

In 10 long-term simulations of the ‘game only’ scenario, the common phenotypic distribution was reached before 20 000 generations in seven simulations, and after 26 000 and 106 000 generations in two others, while one simulation had not converged to this solution when it terminated after 105 000 generations. Thus, variation after 20 000 generations in the 30 simulations in the ‘game only’ scenario (electronic supplementary material, figures S2–S5) was caused partly by a delay in arriving at the common phenotype distribution, and all these deviant simulations were still at a phenotype space with lower fitness (population egg production; electronic supplementary material, figure S2) at the end of the simulation.

### (c) Within-population variation

In all scenarios, the adapted populations contained individual diversity in life-history, physiology, behaviour, global organismic state and genetics (electronic supplementary material, figures S3–S6). All adapted populations displayed continuous and gradual variation in phenotypic traits, such as final body mass, number of times afraid during life, age at death, group size and depth at reproduction (electronic supplementary material, figures S2–S5). In addition, sex differences converged among simulations (electronic supplementary material, figures S2–S5).

**Figure 4.**
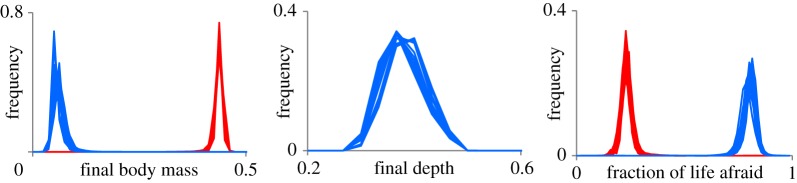
Phenotypic diversity in absence of density-dependent ecological processes. Frequency-distributions of (from left to right) final body mass, depth at reproduction and fraction of life afraid in females (red) and males (blue) in 30 replicate evolutionary runs over 20 000 generations in the ‘no game’ scenario. More examples are given in the electronic supplementary material, figures S2–S5.

Neither frequency-dependence nor environmental variation were needed to maintain this diversity, although variation within populations was lowest in the scenarios where spatial and temporal variation was removed (‘game only’ scenario) or where the behaviour of others did not affect food availability or predation risk, thus making the same vertical trajectory optimal for all individuals (‘no game’ scenario). Even in the ‘no game’ environment, in which there is one single, optimal behavioural solution that fits all individuals, there were differences in final body mass, tendency of being afraid and depth at reproduction (figure 4; electronic supplementary material, figure S2–S5). These differences were caused by coexistence of several alternative neuronal responses to the same environmental stimuli and lead to individual variation in behaviour and, consequently, in phenotypes.

### (d) Among-population variation

**Figure 5.**
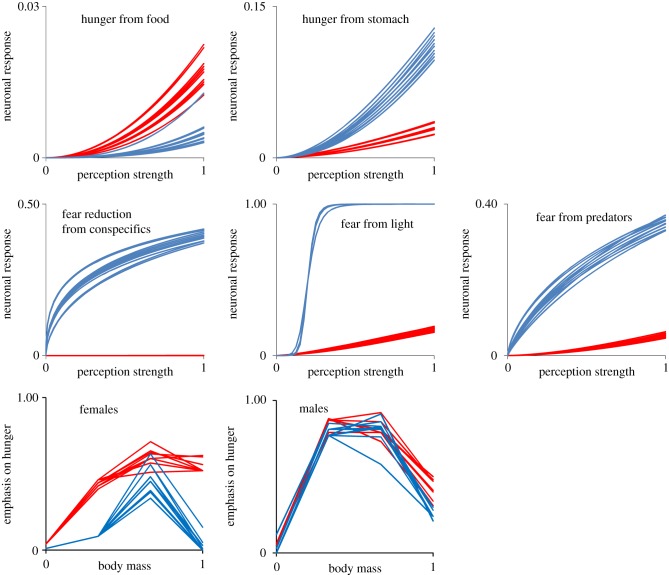
Differences in the neuronal responses (top: neuronal responses in fear; centre: in hunger) and developmental modulators (bottom) in the emotional appraisal, which determines whether the individual is hungry or afraid (in the upper half of figure 1). The 10 most abundant curves are shown for the least (red) and most (blue) frequently frightened population after 120 000 generations among the 10 populations in the ‘full scenario’.

The patterns in behaviours and life histories converged between simulations within each scenario, whereas global organismic state and genetic solutions did not, as shown for the full scenario in figures 2 and 5 and for all scenarios in figure 3 and the electronic supplementary material, figures S2–S5.

While the adaptive solutions at higher levels in the architectural complex (behaviours, physiologies and life histories) were predictable, meaning that each simulation of a scenario produced similar frequency-distributions of parameters for that environment, the solutions at lower levels (genetics and global organismic state) could not be predicted from environmental factors. This is clear from the low CV among populations in figure 3, contrasted with the high CV in global organismic state and genome in the same figure. Such variation among populations was also visible at the level of the neuronal responses and developmental modulators (figure 5), where populations adapted to very similar environments—except for random factors and the evolving gene pool itself—arrived at very different solutions. Individuals from the most frightened population in figure 5 had strong neuronal responses to light intensity and predators, and developmental modulators in females that acted to increase the frequency of fear while conspecifics nearby had a clear calming effect. In the least frightened population from the same scenario, all of these neuronal responses were weaker, and the developmental modulators drove the animals towards hunger.

## 4. Discussion

### (a) The diversifying effect of architecture

Our results suggest that trait architecture has a diversifying effect on trait evolution within a population, and neither environmental variation nor frequency-dependence is a prerequisite for diversity. The flexibility granted by architecture is evident in the increasing variation from life-history traits to genes when one compares across populations. This shows that the critique emphasized by the phenotypic gambit [12] and the behavioural gambit [16] is very relevant, because simplified models without intermediate layers will underestimate natural variability at lower levels of biological organisation, including the genotypic.

The partly random historic path of the evolving gene pool itself becomes important in determining its future [9,40,41], as for other systems when external forcing becomes weak. This is seen in the accumulated differences in what makes the red and blue populations in figure 5 afraid. The strong frequency- and density-dependent selection in the environment in most scenarios leads to the array of co-adapted phenotypes rather than a single best strategy. However, even in the scenarios without these external forces, the populations arrived at a diversity of both phenotypes and genotypes.

### (b) Drivers for diversity

As is true in natural populations, the diversity seen in our study has several causes. *Diversity between phenotypes* is created in each generation by sexual reproduction, where each individual genome is a mix of its parents' genomes and thus may be viewed as a new evolutionary experiment never seen before. Diversity is further increased by numerous stochastic events at the level of the individual [24], which is also important for variation in state-dependent life-history models [7,8]. In addition, a temporally varying fitness landscape, where ephemeral hills and valleys constantly emerge, enables coexistence of multiple strategies and solutions. This is partly caused by frequency-dependent games between strategies [24], through which competition and mutualism affect behaviour, growth and mortality. In addition, a variable environment amplifies gene–environment interactions through shifting periods where some allele combinations prosper and others decline [24].

*Variation increases and predictability decreases from phenotype to genotype* because there is a complex and nonlinear mapping between genotype and phenotype (figure 1). For example, the likelihood of becoming afraid can change through any of the three neuronal responses related to fear or the two related to hunger, or the four developmental modulator genes. Similar nonlinearity applies to other brain configurations, e.g. neural networks [42–45]. This nonlinearity gives rich opportunity for evolvability [18–20] and evolutionary innovations [21–23] at the genetic, neurobiological and emotional levels. The architecture defines a vast space of possible individual and population configurations (e.g. figure 5), as is observed in evo-devo [46,47] and phenotypic plasticity [48].

*Between populations*, the existence or extinction of specific strategies lead to path-dependency [9], whereby a particular population ends up with only one of many possible population configurations at a particular time. The multiple sources for maintenance of biological variation lead to rich variation within and among populations in a wide variety of conditions, and by the different scenario experiments we show that this pattern is quite robust.

Together, these mechanisms yield a unique historic contingency with multiple opportunities for diverging selection among populations. This highlights two sources of biodiversity. Path-dependency gives among-population variation in averages and a potential for higher order diversity, like speciation, whereas within-population variation originates from architecture and may be strengthened by spatio-temporal games among coexisting strategies. As selective freedom is highest for genes, diversity among populations decreases from genes, via emotions, to behaviour, physiology and life-history traits (figure 3 and the electronic supplementary material, figure S1). Experimental studies have found large behavioural differences within and among natural fish populations [49–51] and our work suggests that the genetic, neurobiological and emotional basis for such differences can accumulate even in the absence of long-term environmental differences.

### (c) Higher order effects of architecture

The variation among populations in genetics, neurobiology and emotions, contrasted with the consistency among populations in growth, space use and life history, indicates that a diversity of life forms can adapt to the same environment by evolving different genetic, neurobiological and emotional innovations. It also emphasizes the role of the organismal level of selection [52], as adaptation to the environment does not depend on particular gene pools.

Gould & Lewontin [53] objected to the adaptive phenotype as a ‘Bauplan’. Their argument was that one cannot expect evolution to optimize all sorts of phenotypic characters, as observed by Giske *et al*. [24]. But the Bauplan also comes with evolutionary advantages. One is that architecture-driven diversity among populations in a region may be a mechanism for preadaptation [54,55] and exaptation [56], because this diversity increases the possibility that some of these populations will contain genetic variation that may become important for future adaptability towards new challenges [57]. Another advantage is that architecture by itself enhances genetic variability in the population and thereby reduces the risk that the gene pool will get stuck at a local sub-optimal peak in the fitness landscape.

Architecture will cause individual variation and at the same time population-level phenotypic similarity. Trait architecture makes organisms more constrained than what is usually assumed in optimization theory, in the sense that genetics limit phenotypic plasticity [24]. However, trait architecture also makes populations more diverse and evolvable [18–20]. Thus, it is likely that the emotion system has promoted itself through evolution not only by enhancing the survival of the individual but also through the evolvability of the gene pool. The trait architecture of emotion in animals [28] gives flexible individuals that are able to respond adequately to a wide range of familiar and unfamiliar situations [58,59], and also robust gene pools adaptable to a wide range of new challenges [20]. Trait architecture, and its consequence, path-dependency, may be important factors creating and sustaining diversity, as well as in determining evolutionary winners.

## Supplementary Material

Supplementary figures S1-S5

## Data accessibility

Fortran code is deposited in the Dryad Repository (doi:10.5061/dryad.m6k1r).
