# Supplementary material for: The emotion system promotes diversity and evolvability
Source: Proc Biol Sci. 2014 Sep 22;281(1791):20141096. doi: 10.1098/rspb.2014.1096 (PMC4132677; doi:10.1098/rspb.2014.1096)
Supplement: Supplementary figures S1-S5 [file rspb20141096supp1.docx]

Electronic Supplementary Material to

**The emotion system promotes diversity and evolvability**

Proc R Soc B

July 2014

Jarl Giske^1,2^, Sigrunn Eliassen^1,2^, Øyvind Fiksen^1,2^, Per J. Jakobsen^1^, Dag L. Aksnes^1,2^, Marc Mangel^1,2,3^ & Christian Jørgensen^2,4^

^1^ Department of Biology, University of Bergen, Postboks 7803, 5020 Bergen, Norway

^2^ Hjort Centre for Marine Ecosystem Dynamics, Bergen, Norway

^3^ Center for Stock Assessment Research and Department of Applied Mathematics and Statistics, University of California, 1156 High Street, Santa Cruz, CA 95064, USA

^4^ Uni Computing, Uni Research, Thormøhlensgate 55, 5008 Bergen, Norway

Corresponding author: Jarl Giske: jarl.giske@bio.uib.no

This ESM contains supplementary figures S1-S5.

**Figure S1**. Details of variation within and among populations in scenario experiments. The scenarios (defined in table 1) are (*a*) Full scenario, (*b*) Repetitive environment, (*c*) No game, and (*d*) Game only. Left columns: Within-population diversity among the 30 populations in each scenario, quantified as average within-generation CV, for 88 individual variables (listed below). Coloured areas illustrate average CV and black lines total range among the 30 populations. Right columns: Evolutionary diversity among the 30 populations in the scenario, illustrated as interquartile range (coloured areas) and total range (black lines) of within-population averages for the same variables (normalized against average among populations). The 88 variables are from bottom to top **developmental genes:** developmental modulation genes at birth (1), 1/3 maximum body size (2), 2/3 maximum body size (3), and maximum body size (4) for females and for males (5-8), **genes for GOS (global organismic state)**: the neuronal response genes in hunger from seeing food (9-10), in hunger from stomach (11-12), in fear reduction from conspecifics (13-14), in fear from light (15-16), and in fear from predators (17-18), **genes for behaviour**: the neuronal response genes in attraction to food (19-20) and in avoidance of conspecifics (21-22) when hungry, and in attraction to conspecifics (23-24) and in avoidance of light (25-26) when afraid, **GOS**: number of times afraid for females (27-32) and males (33-38) in six equally long periods through life, **behaviour**: depth (39-44, and 45-50) and number of conspecifics in same depth (51-56, and 57-62) of females and males at six ages through life, **physiology**: stomach fullness (63-68, and 69-74) and body mass (75-79, and 80-84) of females and males at six and five ages through life, respectively, and **life history**: age at death (85-86) and final body mass of surviving females and males (87-88).

**Figure S2.** Evolution in populations in the 4 scenarios. 30 replicate evolutionary runs over 20,000 generations of population total egg production (left), mortality rate (centre), and fraction of time being afraid (right). In the Game only scenario, only 17 of the 30 populations located the highest fitness plateau, while the 4 red and 9 blue populations did not. In the 3 other scenarios, males are blue, females red, and both genders together are black.

**Figure S3**. Phenotypic diversity within and among populations in the 4 scenarios. Frequency-distributions in the 40 last generations of 30 replicate evolutionary runs over 20,000 generations of final body mass (left), death rate through life (centre), and depth at reproduction (right). In the Full scenario, the 40 last “standard environment” generations (table 2) are used. In the Game only scenario, only the populations included in figures 3 and S1 are shown. Males are blue, females red.

**Figure S4**. Phenotypic diversity within and among populations in the 4 scenarios. Frequency-distributions the 40 last generations in 30 replicate evolutionary runs over 20,000 generations of average number of neighbours in the same depth each age (left), fraction being afraid each age (centre), and number of fish in same depth (right). Four events of schools of predators attacking all populations in the same time steps are visible as peaks in fear and number of neighbours. In the Full scenario, the 40 last “standard environment” generations (table 2) are used. In the Game only scenario, only the populations included in figures 3 and S1 are shown. Males are blue, females red.

**Figure S5**. Phenotypic diversity within and among populations in the 4 scenarios. of 30 replicate evolutionary runs over 20,000 generations: Frequency-distributions of fraction of life being afraid (left), average depth each time step during the second last diel cycle (centre), and available stomach capacity through life (right). In the Full scenario, the 40 last “standard environment” generations (table 2) are used. In the Game only scenario, only the populations included in figures 3 and S1 are shown. Males are blue, females red.
